# Single-nuclei transcriptomics enable detection of somatic variants in patient brain tissue

**DOI:** 10.1038/s41598-023-27700-6

**Published:** 2023-01-11

**Authors:** Sydney E. Townsend, Jesse J. Westfall, Jason B. Navarro, Daniel C. Koboldt, Elaine R. Mardis, Katherine E. Miller, Tracy A. Bedrosian

**Affiliations:** 1 Institute for Genomic Medicine, The Abigail Wexner Research Institute at Nationwide Children’s Hospital, Columbus, OH 43215 USA; 2 Biomedical Sciences Graduate Program, College of Medicine, The Ohio State University, Columbus, OH 43210 USA; 3 Department of Pediatrics, College of Medicine, The Ohio State University, Columbus, OH 43210 USA; 4 Department of Neurosurgery, College of Medicine, The Ohio State University, Columbus, OH 43210 USA

**Keywords:** Transcriptomics, Mutation, Genetics of the nervous system

## Abstract

Somatic variants are a major cause of human disease, including neurological disorders like focal epilepsies, but can be challenging to study due to their mosaicism in bulk tissue biopsies. Coupling single-cell genotype and transcriptomic data has potential to provide insight into the role somatic variants play in disease etiology, such as by determining what cell types are affected or how the mutations affect gene expression. Here, we asked whether commonly used single-nucleus 3’- or 5’-RNA-sequencing assays can be used to derive single-nucleus genotype data for a priori known variants that are located near to either end of a transcript. To that end, we compared performance of commercially available single-nuclei 3’- and 5’- gene expression kits using resected brain samples from three pediatric patients with focal epilepsy. We quantified the ability to detect genetic variants in single-nucleus datasets depending on distance from the transcript end. Finally, we demonstrated the ability to identify affected cell types in a patient with a *RHEB* somatic variant causing an epilepsy-associated cortical malformation. Our results demonstrate that single-nuclei 3’ or 5’-RNA-sequencing data can be used to identify known somatic variants in single-nuclei when they are expressed within proximity to a transcript end.

## Introduction

Somatic variants are post-zygotic DNA alterations that can be acquired beginning in embryonic development and over the course of an individual's lifetime. In contrast to germline variants, somatic variants lead to mosaicism across different tissues and cell types^1,2^. Somatic variants are recognized as a cause of human disease, including cancer, vascular and brain malformations, and focal epilepsies^3^. Nevertheless, it has been challenging to study somatic variants in patient biopsies due to their mosaic nature. Some disease-causing somatic variants are present in less than 1% of cells, which makes it difficult to discern their molecular effects or cell-type-specificity using bulk tissue assays^4,5^. Therefore, coupling genotyping and transcriptomics from single cells has potential to reveal new insights into cell-specific disease etiology.

Genotyping from single-cell transcriptomic data is possible with several caveats. First, the gene containing a variant of interest must be expressed within the dataset. Loss-of-function variants that cause nonsense-mediated decay of transcripts are not ideal candidates for genotyping in transcriptomic data. Second, the choice of single-cell RNA-sequencing methodology is critical. Sequencing full-length transcripts enables detection of variants located anywhere within a transcript; however, some whole transcript approaches are limited by throughput, making it challenging to genotype large numbers of cells as is required to detect low frequency mosaic variants. Droplet-based single-cell 3’ and 5’-RNA-sequencing methods produce limited coverage restricted to the transcript ends; however, they provide much higher throughput at lower cost^6^.

For variants of interest that reside near to either transcript end, single-cell genotype information could be easily obtained without any additional cost or labor from commonly used single-cell 3’- or 5’-RNA-sequencing kits. Several studies have investigated the utility of such an approach for single cells derived from cancer patients; however, the approach has not yet been applied systematically to patient brain tissue, from which nuclei are most easily accessible^7,8^. Further, few studies have directly compared single-nuclei 3’ versus 5’-RNA-sequencing data to understand whether the assays can be directly interchanged when desired for identifying specific somatic variants. We investigated the feasibility of identifying somatic variants in single-nuclei 3’ or 5’-RNA-sequencing data, by comparing performance of commercially available 3’- and 5’- gene expression kits using brain tissue from three pediatric patients with focal epilepsies. We tested our ability to identify variants on a single-nuclei basis from these datasets depending on the distance of a variant from the transcript end. Finally, we demonstrated a successful example of single-nuclei genotyping from 5’-RNA-sequencing data that enabled us to determine the affected cell types in a patient with a *RHEB* somatic variant.

## Results

### Study overview

**Figure 1:**
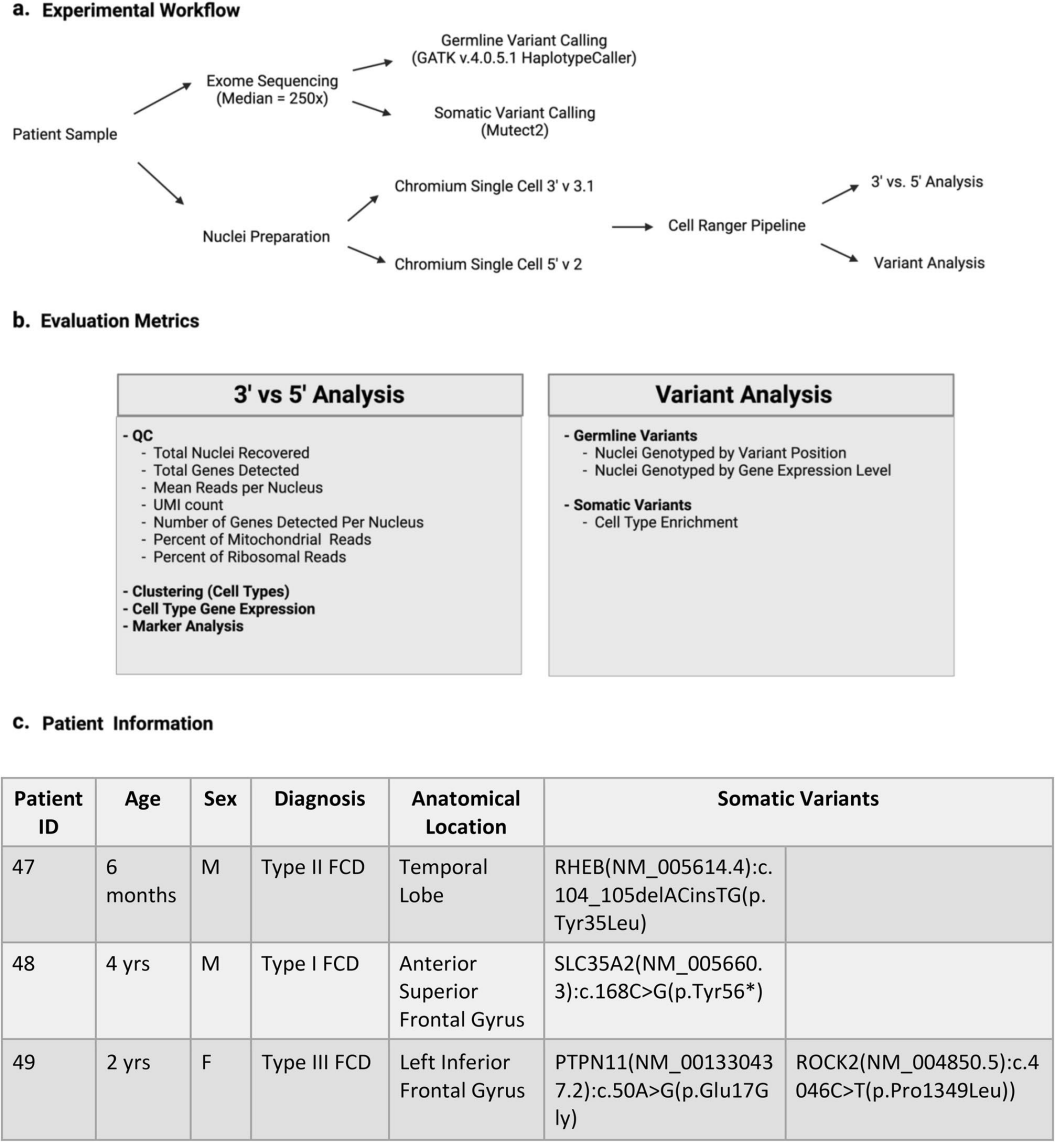
Study overview. (**a**) Patient samples were divided for variant calling from bulk exome sequencing and for single-nuclei 5’- and 3’-RNA-seq. (**b**) snRNA-seq datasets were evaluated for comparative performance metrics and detection of variants previously identified in exome sequencing. (**c**) Biomaterial was obtained from the temporal or frontal lobe of three patients in an IRB approved protocol.

We established a workflow to compare performance between the 10 × Genomics Chromium NextGEM Single-Cell 3’ and 5’ gene expression kits and evaluate detection of known germline and somatic variants in the resulting datasets (Fig. 1). For these analyses, we obtained frozen resected brain tissue from three pediatric patients treated for focal epilepsy and enrolled in an IRB-approved research protocol. In a previous study, germline and somatic variants were identified in bulk tissue samples via exome sequencing analysis^9^. In this study, we performed single-nuclei RNA-sequencing (snRNA-seq) on remaining material cut from the same tissue section (Fig. 1a). To evaluate performance of the 3’ versus 5’ gene expression kits, we compared QC metrics, gene expression, cell type clustering, and marker gene efficacy. For variant analysis we evaluated the detection rate of both germline and somatic variants depending on distance from the transcript end and expression level of each gene of interest (Fig. 1b). Finally, as a proof-of-concept, we identified cell types expressing a disease-causing *RHEB* variant using single-nuclei 5’-RNA-seq data (Fig. 1c).

### Consistent QC and gene expression across kits

**Figure 2:**
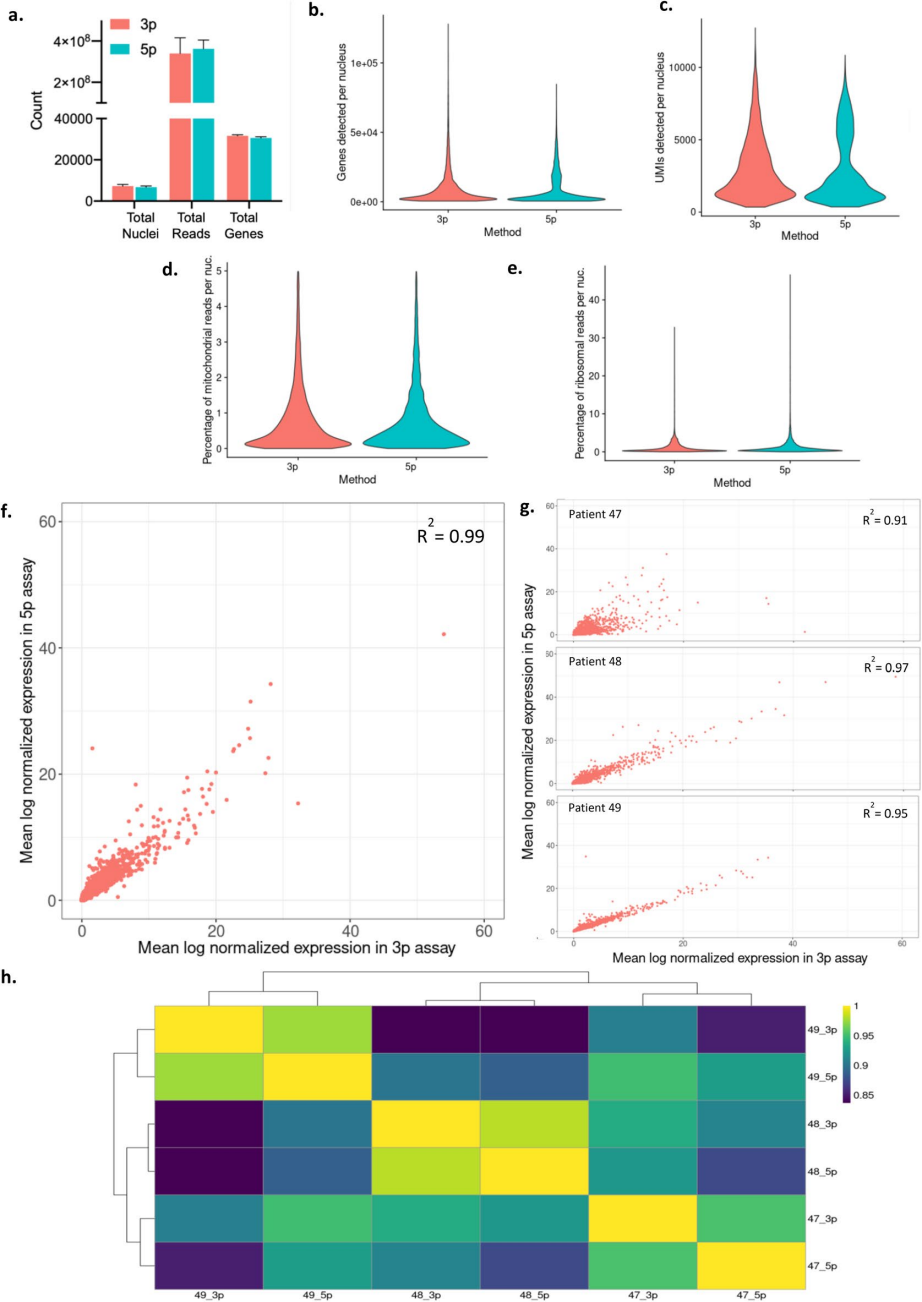
Consistent sensitivity and gene expression across kits. (**a**) 5’ and 3’-RNA-seq libraries were sequenced to similar depth with similar number of nuclei captured. (**b,c**) Number of UMIs and genes detected per nucleus were similar for each kit. (**d,e**) Quality metrics (percent mitochondrial and ribosomal reads per nucleus) were similarly low for each kit. (**f,g**) Mean log-normalized gene expression values were highly correlated by kit both on average and on a per-patient basis. (**h**) Correlation matrix showing high concordance of gene expression in 3’ versus 5’ datasets.

We generated data from 41,953 nuclei in total from three patient samples (µ = 7,000 nuclei per sample), sequenced to equivalent depth with single-nuclei 3’ or 5’-RNA-seq kits (Fig. 2a). Both kits had similar sensitivity in detecting RNA molecules, yielding a similar number of UMIs and genes detected per nucleus (Fig. 2b,c). The quality of captured nuclei was also similar, as we observed a similar low proportion of mitochondrial and ribosomal genes, which is a metric that has been used to detect low-quality or stressed cells (Fig. 2d,e)^10^. We repeated these comparisons on a patient specific level and found similar results (Fig. S1). Next, we compared average gene expression by kit, which we expected to be similar as both sets of nuclei were isolated from the same original tissue section. Indeed, the mean log-normalized gene expression values by kit were highly correlated on average (R^2^ = 0.99) (Fig. 2f), as well as on a patient-specific basis (Fig. 2g,h).

### Similar cell type distribution and proportion

**Figure 3:**
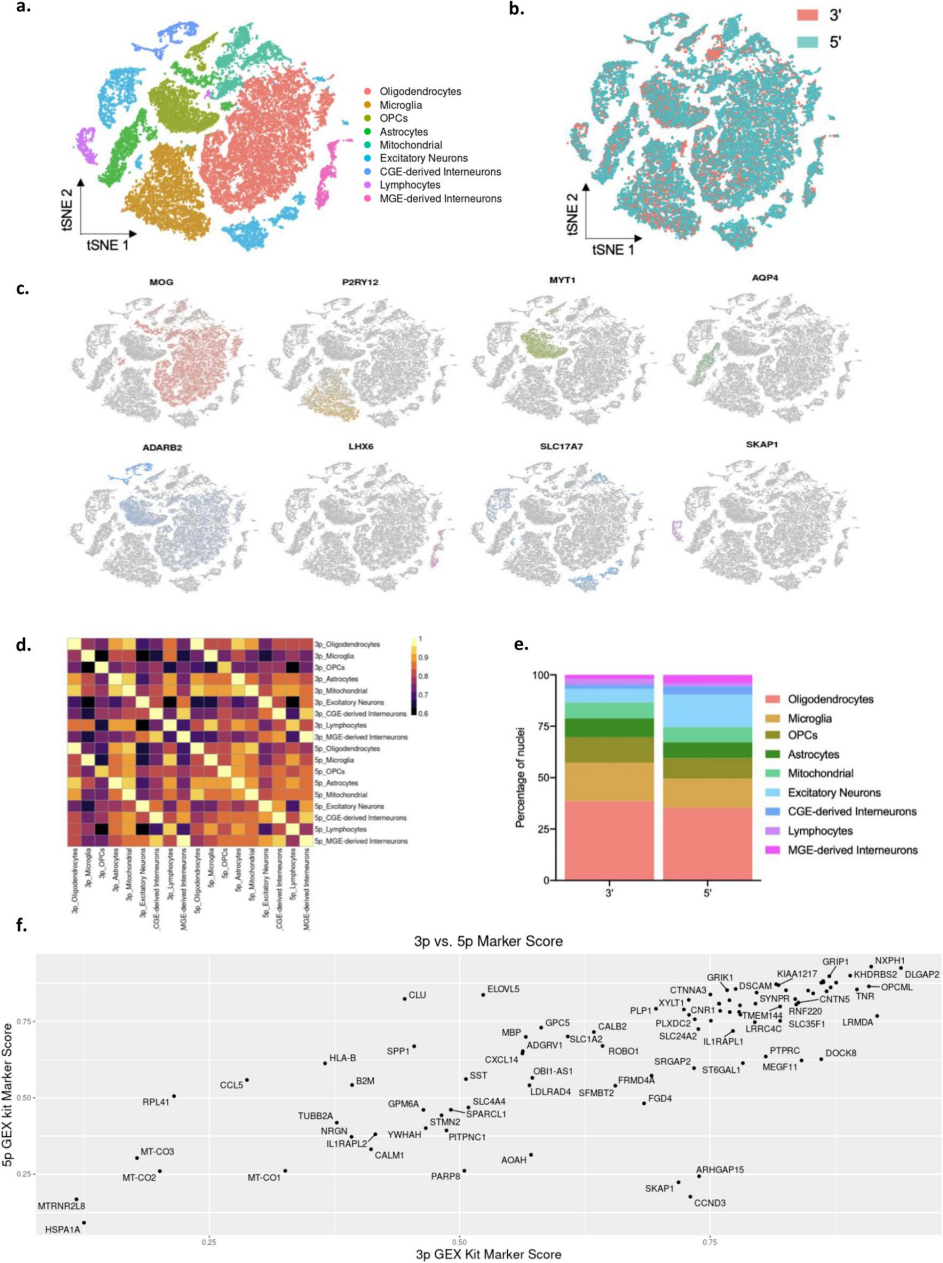
Similar cell type distribution and proportion. (**a**) Nuclei integrated together from all six libraries clustered into nine cell type clusters. (**b**) Distribution of nuclei across clusters was similar for those sequenced with the 5’ or the 3’ kits. (**c**) Canonical cell type markers used to identify the nine cell type clusters. (**d**) Correlation matrix of gene expression by kit for each cell type. (**e**) Percentage of nuclei in each cell type cluster by kit. (**f**) Marker gene efficacy is similar for each capture method.

As cell type identification is a major goal of single-cell RNA-sequencing experiments, we determined whether the two kits detected a similar distribution and proportion of cell types. We integrated all six datasets and clustered nuclei by their gene expression profiles, performing annotation by examining expression of known marker genes (Fig. 3a). In general, we observed a similar distribution of nuclei across both kits (Fig. 3b,c). To determine the similarity of gene expression within the detected cell types by kit, we performed pairwise correlations between kits for each of the cell type clusters using average gene expression values. Cell types identified in each kit were highly similar, as the lowest correlation between two cell types was 0.95 (Fig. 3d). We then quantified the number of nuclei per cell type for each capture method and found similar numbers of nuclei per cell type for each kit. There were slight differences in the proportion of excitatory neuron populations and MGE-Derived interneurons (Fig. 3e). This could be due to random variation in cell type capture or a difference in the efficacy of marker genes between kits. To assess this possible difference in cell type markers for each kit we utilized a technique by Brekken et al.^11^ to calculate a marker gene efficacy score (Fig. 3f). We found that most cell type markers had similar efficacy for both 3’ and 5’-RNA-seq data sets. Marker genes that were high-scoring in one dataset but not in the other (ARHGAP15, SKAP1, and CCND3) can be explained by the low number of nuclei present in their respective cell type clusters (Fig. 3f, S2).

### Detection of germline variants in snRNA-seq data

**Figure 4:**
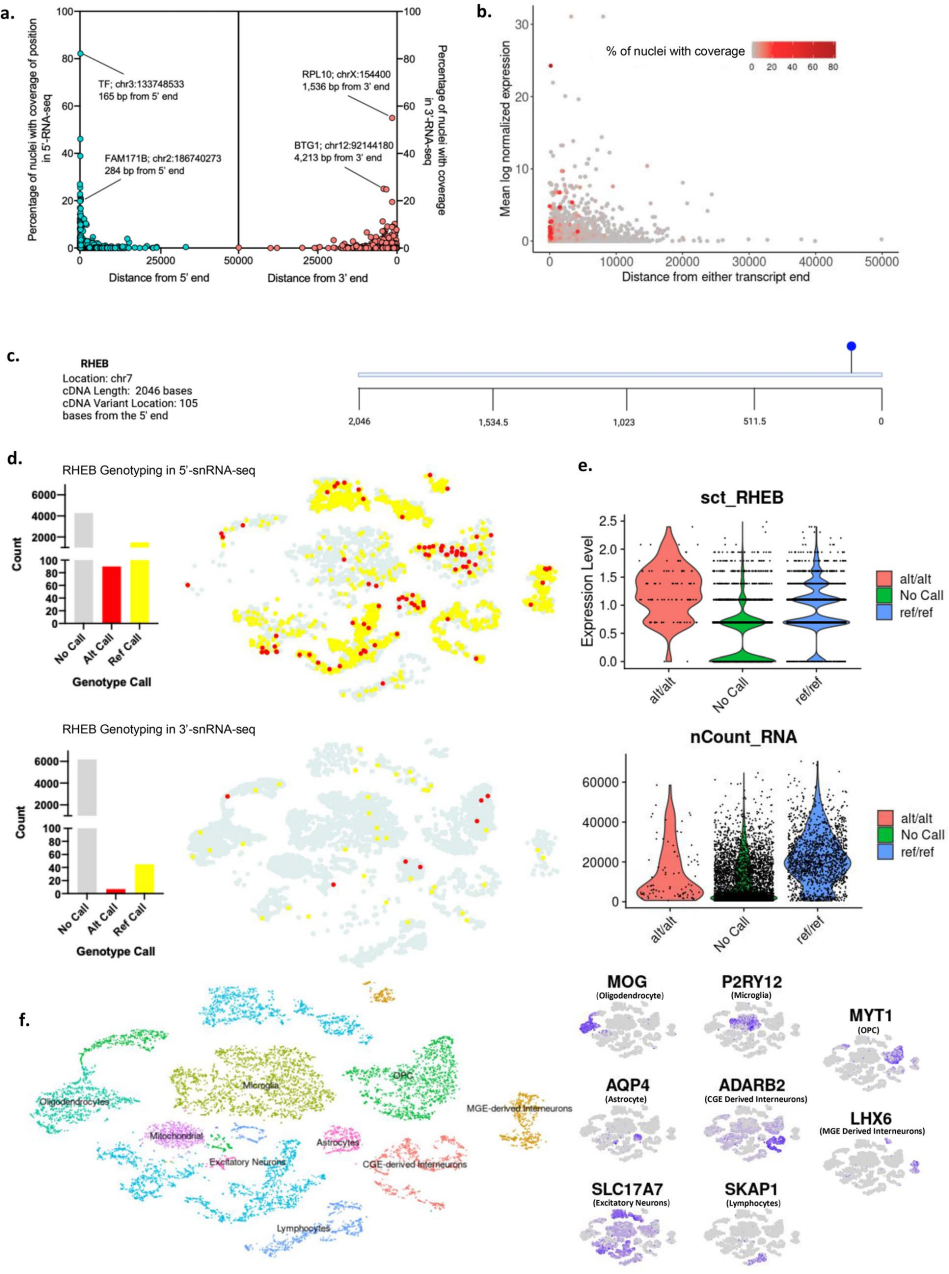
Detection of genetic variants in single-cell transcriptomes. (**a**) Germline variants were called for each patient from exome data and the proportion of cells with coverage of germline variant positions in single-nuclei transcriptomic data was plotted versus each variant’s proximity to the transcript end. Noted are variant positions with high coverage but greater than expected distance from the 5’ or 3’ transcript end. All calculations were done on a patient-specific basis. (**b**) Percentage of nuclei with coverage of each germline variant position by distance from transcript end and expression level of the gene. (**c**) *RHEB* variant position. (**d**) The proportion of *RHEB* genotype calls in nuclei from patient 47 captured via the 3’ or 5’ kit. (**e**) SCT normalized gene expression for RHEB shown at a single-nucleus level for each genotype call (top). Number of unique RNA molecules detected per nucleus for each genotype call (bottom). (**f**) The distribution of *RHEB* genotype calls within cell type clusters from the 3’or 5’ datasets and examples of cell type marker genes expressed within clusters of the patient 47 dataset.

Having shown that samples processed via either kit have similar gene expression and cell type distribution, we next aimed to characterize our ability to detect genetic variants in snRNA-seq datasets. For this analysis, we used a set of exonic germline variant positions previously identified in these tissues via bulk tissue exome sequencing^9^. We gauged the detection sensitivity of snRNA-seq by looking at coverage of these positions in our datasets. First, we asked how distance from a transcript end (3’ or 5’) affects the rate of detection for the variant positions in snRNA-seq data. The 10 × Genomics Chromium Next GEM Single-Cell 3’ and 5’ gene expression kits sequence 91 and 90 bp from the captured transcript end respectively. 58.2% of positions within 100 bp of the 5’ or 3’ transcript end were identified in at least 5% of nuclei in their respective dataset. However, we found several examples in which we had coverage of positions hundreds or thousands of base pairs from the transcript end (Fig. 4a). Detection of positions far from the transcript end could be a result of mispriming, which has been previously shown to occur with these technologies^12^. During reverse transcription, a poly dT primer is used to target the Poly-A tail of mRNA molecules. If there are any repeated A sequences within the body of the transcript, those sequences could be misprimed and initiate reverse transcription instead. An example is RTN4 p.Ser440Asn. It is a variant position that should not be detected due to its location near the middle of the transcript, yet it was detected in ~ 25% and 12% of nuclei from our 5’ and 3’-RNA-seq datasets. Surrounding this gene we found repeat A-sequences that suggest internal poly-A mispriming may have lead to the observed coverage of this position (Fig. S3). 45.1% of positions located within 1,000 bp of a transcript end had coverage in at least 5% of nuclei, suggesting mispriming occurs to a significant extent in 10 × Genomics single-nuclei gene expression data. On the other hand, there were positions that fell within the 91 bp constraint but had little to no representation in our snRNA-seq data. Therefore, we queried the average log normalized expression of each gene and correlated this to distance from the transcript end and proportion of nuclei genotyped (Fig. 4b). Positions near the transcript end that had little representation in our snRNA-seq data were mostly not highly expressed in our dataset. Overall, 77.2% of positions in genes moderately expressed (log normalized abundance > 5; range 0–31) were detected in at least 5% of cells, whereas only 36.7% of positions in genes with log normalized abundance > 0.1 were detected. Finally, to assess the detection rate of false positives, we looked for germline variants not expected to be found in any given patient. For this test, we used germline exonic variants called by GATK haplotype caller that were absent in gnomAD (total = 432). The rationale for using variants absent from gnomAD is to ensure they aren't common germline variants that could be seen in another patient. We manually confirmed no overlap of variants across the three patients. We observed only two false positive “alt” calls (false positive rate < 0.5%). In both cases, the false positive call was only detected in a single nucleus.

### Successful detection of RHEB somatic variant

From our whole exome sequencing data, we previously identified candidate disease-causing variants in our patients^9^. There was at least one candidate somatic variant present within each patient. One patient diagnosed with Type II focal cortical dysplasia (FCD) had a somatic variant in *RHEB,* a gene that is known to play a major role in the mTOR signaling pathway and has been altered in patients with FCD^13^. This particular variant was located within the first 105 nucleotides from the transcription start site, making it an ideal candidate for genotyping in single-nuclei 5’-RNA-seq data (Fig. 4c). We used VarTrix (GitHub–10XGenomics/vartrix: Single-Cell Genotyping Tool) to identify reference- and variant-supporting reads in individual nuclei and then quantified the number of variant calls (Alt), reference calls (Ref), and the absence of coverage (No call) for each chemistry. The *RHEB* variant was expressed in about 5% of cells in total, which is close to the 11.40% VAF detected by bulk exome sequencing. As expected, we observed a higher number of genotyped nuclei within our 5’ dataset (27%) versus the 3’ dataset (0.7%) because of the variant’s proximity to the 5’ transcript end (Fig. 4d). Coverage and gene expression are also important determinants of variant identification. A large proportion of nuclei in the No Call group had zero expression of RHEB and a lower overall number of RNA molecules detected per nucleus (Fig. 4e). The small number of nuclei genotyped in the 3’ dataset may be caused by an A repeat sequence observed just upstream of the variant position. We then wanted to determine how these variant calls were distributed across cell types within our datasets. Most Alt calls were enriched in Microglia, Excitatory Neurons, Astrocytes, Oligodendrocytes, and Oligodendrocyte Precursor Cells at VAFs of 5%, 3%, 34%, 43%, and 31%, respectively (Fig. 4f). As most cell types expressed the *RHEB* variant, including cell types from distinct developmental lineages (i.e., microglia from the mesoderm and neurons from the ectoderm), we reasoned that this somatic variant arose before gastrulation. We repeated the analysis on candidate variants for the other two patients, but in one case the gene was not expressed highly enough to obtain meaningful genotyping data and in the other case the variant was too far from the transcript end (Fig. S4).

## Discussion

Our results show comparable performance of 10 × Genomics 3’ and 5’ gene expression kits in quality control metrics, gene expression, and cell type recovery when directly comparing the same patient samples. Our observations are consistent with a previous study that demonstrated high transcript and gene detection sensitivity of 10 × Genomics Single-cell 3’-RNA-seq v.3.0 and 5’-RNA-seq v.1 kits compared to other scRNA-seq methods^14^. We expanded on these findings by investigating the most recent 5’ v.2 and 3’ v.3.1 kits and found similar numbers of transcripts and genes detected per cell, as well as similar gene expression and cell type recovery. The similar kit performance suggests that it would be appropriate to decide which chemistry to use based on targeted variant location, in cases where single-nuclei genotyping is desired as we demonstrated here. We were able to show the successful detection of genetic variants from 10 × Chromium gene expression datasets, provided that the variant is (a) expressed and (b) located near to a transcript end or in proximity to an alternative polyA capture site. We demonstrated how the ability to detect somatic variants in single cell transcriptomic data can give insight into the affected cell types within an epilepsy-associated malformation of cortical development.

We chose to use VarTrix from 10 × Genomics to identify reference and variant-supporting reads within our single-nuclei transcriptomic datasets because it provides a streamlined user experience when starting with CellRanger output files. Our results confirmed that VarTrix can identify a priori known variants in snRNA-seq data with a low false positive rate of < 0.5%. VarTrix is one of many tools developed for this purpose. Notably, SCReadCounts not only tabulates read counts at known variant positions, but also has a discovery mode for identifying novel somatic variants in single-cell RNA-seq data. We replicate the results of Prashant et al. using VarTrix and obtain very similar results (https://github.com/bedrosian-lab/5p3p_genotyping)^15^. SCMut is a tool that can discover somatic variants in single-cell transcriptomes and control for false positives^16^. Selection of tool will depend on the needs of each specific study.

Our analysis of the *RHEB* somatic variant provides an example of the utility of genotyping from single-nuclei transcriptomic data. This analysis enabled us to determine that the *RHEB* variant is enriched, but not restricted to Microglia, Neurons, Astrocytes, Oligodendrocytes, and Oligodendrocyte Precursor Cells. As microglia and other brain cell types arise from different germ layers in development, this implies that this post-zygotic variant is acquired before gastrulation occurs. This finding is meaningful because it provides a model of how this disease-causing variant arose in development and it suggests the variant could potentially be found in other tissues of the patient besides brain with yet to be determined consequences.

Genotyping from single-cell 5’ or 3’ gene expression data will always be limited in utility to specific kinds of variants. Other groups have attempted to extend on the 10 × Genomics platform to obtain improved genotype or isoform information from droplet based single-cell RNA-sequencing data by incorporating additional sequencing of leftover barcoded cDNA from the 10 × Genomics protocol^8,17^. One method that we recently published leveraged long-read sequencing of the leftover full-length barcoded cDNA fraction to genotype somatic variants deep in the *PTEN* gene^18^. A second method developed by Nam et al. performs amplicon sequencing of positions of interest in the leftover cDNA fraction^8^. Both methods have been successful in correlating genetic information to scRNA-seq datasets; however, there are some aspects that make these methods less user friendly. The first is the utilization of other sequencing methods. This can add additional cost and effort to an already costly technology. Another caveat of using other sequencing methods is that long-read sequencing currently has lower throughput than short-read sequencing. This limitation may be addressed as sequencing technologies advance.

Another limitation mentioned above is mispriming that occurs when repeated A sequences are present within the transcript. These A-repeat sequences can serve as alternative polyA capture sites and can initiate reverse transcription^12^. Depending on their location, they can lead to unexpected sequencing coverage far from a transcript end. While mispriming can result in unwanted off-target effects, it can also allow for variants to be sequenced far from the transcript end. This of course is not a factor that can be controlled. But the presence of an alternative polyA capture site could increase the chances for representation of a genetic variant regardless of its position in the transcript.

Here, we provided a direct comparison of single-nuclei 5’ versus 3’-RNA-seq using parallel patient samples. We also benchmarked our ability to detect genetic variants in 10 × Chromium gene expression datasets. This approach could allow researchers to gain genotype information from datasets without the need for additional sequencing methods in some cases. This allows for more insights to be gathered with fewer resources. This increased information can allow for the production of new insights that can inform the field on the role somatic variants have in different human diseases.

## Methods

### Tissue acquisition and ethical considerations

Tissue samples were derived from surgically resected brain tissue obtained from three pediatric patients enrolled in a research protocol (IRB18-00786) approved by the Nationwide Children’s Hospital Institutional Review Board. All methods were performed in accordance with the appropriate guidelines and regulations. As patients were under the legal age of consent, informed consent was obtained from parent/legal guardians prior to the start of this study. Details of patient enrollment, sample collection, exome sequencing, and variant calling have been previously published^9^.

### Nuclei preparation and sequencing

Snap frozen samples were stored at -80 °C until use. Briefly, tissue was dissociated in a glass dounce homogenizer and then nuclei were washed and stained with Hoechst dye, as previously described^18^. Approximately 15,000 Hoechst-positive nuclei per sample were sorted on a BD Influx cell sorter directly into master mix from the 10 × Genomics Next GEM Single-Cell 3’ or 5’ reagent kits. Reverse transcriptase was added to the master mix and reactions were loaded into a 10 × Genomics Chromium Controller for single-nuclei capture. Libraries were constructed in accordance with the 10 × Genomics Chromium Next GEM Single-Cell 3’ Reagent kit v.3.1 or the Chromium Single-Cell 5’ Reagent kit v.2. Libraries were sequenced on an Illumina NovaSeq 6000 instrument to generate paired-end sequencing data.

### Data pre-processing and quality metrics

Sequencing data were processed using the 10 × Genomics Cell Ranger v.6 analysis pipeline. Generation of fastq files and data preprocessing, including alignment, filtering, barcode counting and UMI counting, were performed using the Cell Ranger mkfastq and count commands following the default parameters. Number of nuclei, number of genes detected, and total reads per library were obtained from Cell Ranger count. Downstream analysis was performed using Seurat v.4 for R^19^. The VlnPlot function was used to visualize number of UMIs and number of genes detected per nucleus. Low-quality nuclei with greater than 5% mitochondrial reads were filtered out. Doublets were identified using DoubletFinder for R and then visualized on FeaturePlots in Seurat and excluded from the datasets before further analysis^20^.

### Single cell RNA-seq data analysis

Normalization and variance-stabilization of feature-barcode matrices was performed using the SCTransform function of Seurat^21^. All six libraries were integrated using the SCTransform method. Dimensionality reduction was performed using principal component analysis and the distance matrix was organized into a K-nearest neighbor graph, partitioned into clusters using Louvain algorithm, and clusters were visualized on a tSNE plot^22,23^. Top differentially expressed genes representing each cluster were found using FindAllMarkers. Cell types were annotated by inspection of canonical marker genes. Nine major cell types were identified: Microglia, Lymphocytes, MGE-Derived Interneurons, CGE-Derived Interneurons, Astrocytes, Oligodendrocytes, Oligodendrocyte Precursor Cells (OPCs), Excitatory Neurons, and Mitochondrial (a cluster of expressing a high proportion of mitochondrial reads).

### Marker gene scoring

To assess the ability of each marker gene to differentiate cell types in 5’ versus 3’ data, we calculated a marker score as previously described^11^. We selected the top 10 marker genes (highest average log2 fold change) for each cell type cluster and then calculated separately for 5’ and 3’ datasets the proportion of cells in each cluster expressing each marker gene at greater than 1 count per million (CPM). Next, we calculated a marker score as the sum of the squared differences in proportions divided by the sum of the differences in proportions. The resulting score (ranging from 0 to 1) represents the specificity of the marker gene, with zero reflecting evenly distributed expression or no expression across all clusters, and one representing perfectly binary expression in the marked cluster.

### Gene expression comparison

To compare overall gene expression by capture method, we normalized raw counts for each gene to read depth (per nucleus), scaled by 10,000, log-transformed using the natural log, and calculated average expression in 5’ versus 3’ data. The analysis was repeated for patient-specific pairs of data. To compare gene expression by capture method for each cell type cluster, we repeated the analysis averaging expression on a per cluster basis and generated a distance matrix using the cor function in base R.

### Variant detection in single-nuclei

For each patient, germline and somatic variation was previously detected from whole exome sequencing analysis of resected brain tissue as published [9(15]. To determine representation of each variant at a patient-specific level in the single-nuclei datasets, we used Vartrix software (GitHub—10XGenomics/vartrix: Single-Cell Genotyping Tool) to generate a call set identifying each nucleus as expressing the variant or reference allele. The calls were joined by cell barcode to each Seurat object’s metadata slot for visualization and to calculate a proportion of cells expressing each variant. The distance of each variant to the transcript end was calculated using MANE transcript IDs and the function “transcriptLengths” from the R package “GenomicFeatures”^24^. Further filtering was done to remove intronic variants from our datasets as we observed some intronic regions were sequenced as a byproduct of WES.

## Supplementary Information

Supplementary Information.

## Data Availability

The datasets generated and/or analysed during the current study are available in the github repository, https://github.com/bedrosian-lab/5p3p_genotyping, along with a tutorial notebook. The single cell RNA-seq data presented in this publication have been deposited in NCBI’s Gene Expression Omnibus (GEO) and are accessible through GEO Series accession number GSE210670. All other data is available upon reasonable request.

## References

[1] Moore L (2021). The mutational landscape of human somatic and germline cells. Nature.

[2] Milholland B (2017). Differences between germline and somatic mutation rates in humans and mice. Nat. Commun..

[3] Poduri A (2013). Somatic mutation, genomic variation, and neurological disease. Science.

[4] Veltman JA, Brunner HG (2012). De novo mutations in human genetic disease. Nat. Rev. Genet..

[5] Biesecker LG, Spinner NB (2013). A genomic view of mosaicism and human disease. Nat. Rev. Genet..

[6] Ziegenhain C (2017). Comparative analysis of single-cell RNA sequencing methods. Mol. Cell.

[7] Petti AA (2019). A general approach for detecting expressed mutations in AML cells using single cell RNA-sequencing. Nat. Commun..

[8] Nam AS (2019). Somatic mutations and cell identity linked by genotyping of transcriptomes. Nature.

[9] Bedrosian TA (2021). Detection of brain somatic variation in epilepsy-associated developmental lesions. Epilepsia.

[10] Ilicic T (2016). Classification of low quality cells from single-cell RNA-seq data. Genome Biol..

[11] Bakken TE (2018). Single-nucleus and single-cell transcriptomes compared in matched cortical cell types. PLoS ONE.

[12] Nam DK (2002). Oligo(dT) primer generates a high frequency of truncated cDNAs through internal poly(A) priming during reverse transcription. Proc. Natl. Acad. Sci. USA.

[13] Lee WS (2021). Gradient of brain mosaic RHEB variants causes a continuum of cortical dysplasia. Ann. Clin. Transl. Neurol..

[14] Yamawaki TM (2021). Systematic comparison of high-throughput single-cell RNA-seq methods for immune cell profiling. BMC Genomics.

[15] Prashant NM (2021). SCReadCounts: Estimation of cell-level SNVs expression from scRNA-seq data. BMC Genomics.

[16] Vu TN (2019). Cell-level somatic mutation detection from single-cell RNA sequencing. Bioinformatics.

[17] Gupta I (2018). Single-cell isoform RNA sequencing characterizes isoforms in thousands of cerebellar cells. Nat. Biotechnol..

[18] Koboldt DC (2021). PTEN somatic mutations contribute to spectrum of cerebral overgrowth. Brain.

[19] Hao Y (2021). Integrated analysis of multimodal single-cell data. Cell.

[20] McGinnis CS, Murrow LM, Gartner ZJ (2019). DoubletFinder: Doublet detection in single-cell RNA sequencing data using artificial nearest neighbors. Cell Syst..

[21] Hafemeister C, Satija R (2019). Normalization and variance stabilization of single-cell RNA-seq data using regularized negative binomial regression. Genome Biol..

[22] Maaten LVD, Hinton G (2008). Visualizing Data using t-SNE. J. Mach. Learn. Res..

[23] Traag VA, Waltman L, van Eck NJ (2019). From louvain to leiden: Guaranteeing well-connected communities. Sci. Rep..

[24] Lawrence M (2013). Software for computing and annotating genomic ranges. PLoS Comput. Biol..

