# Supplementary material for: Single-nuclei transcriptomics enable detection of somatic variants in patient brain tissue
Source: Sci Rep. 2023 Jan 11;13:527. doi: 10.1038/s41598-023-27700-6 (PMC9834227; doi:10.1038/s41598-023-27700-6)
Supplement: Supplementary file 1 — Supplementary Information. [file 41598_2023_27700_MOESM1_ESM.pdf]

Supplemental Figure 1

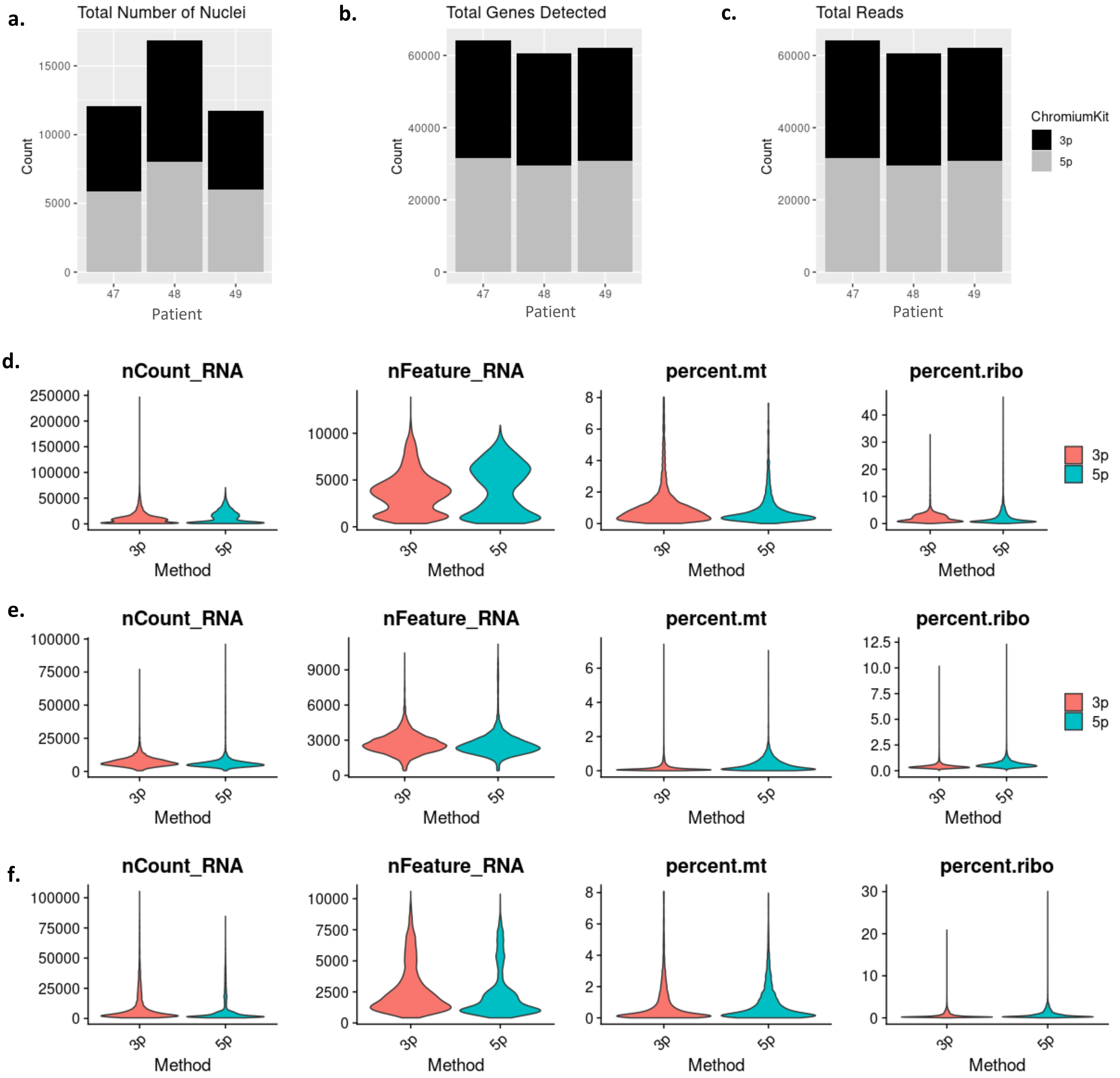

Figure S1: 5’ and 3’ kits perform comparably. (a-c) Total number of nuclei, genes detected, and reads detected for each sample. (d) Number of UMIs per nuclei (nCount_RNA), number of genes per nuclei (nFeature_RNA), percent mitochondrial reads per nucleus (percent.mt), and percent ribosomal reads per nucleus (percent.ribo) for each sample.

Supplemental Figure 2

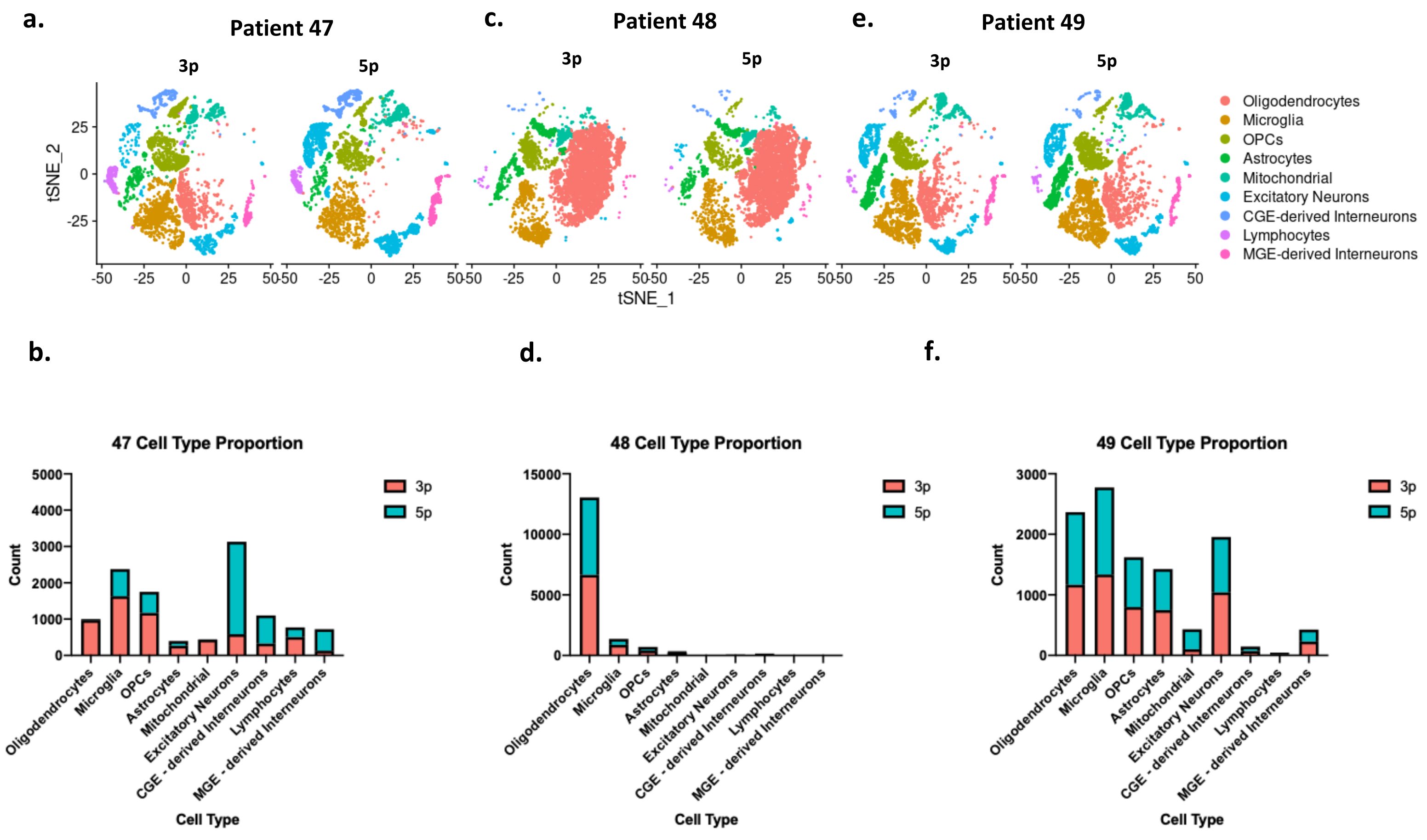

Figure S2: Similar cell type proportion and distribution between kits. Comparison of the distribution and proportion of cell types across patient datasets analyzed with both the 3' and 5' kits. (a, b) Patient 47, (c,d) Patient 48, (e,f) Patient 49.

Supplemental Figure 3

a.

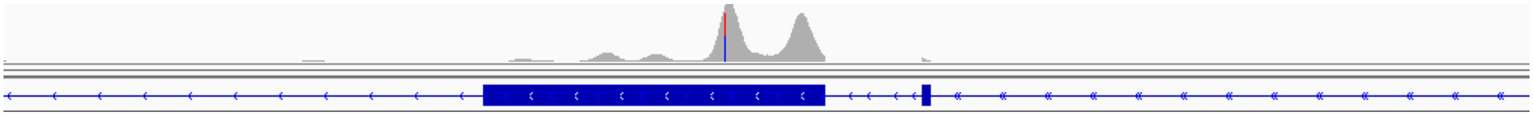

b.

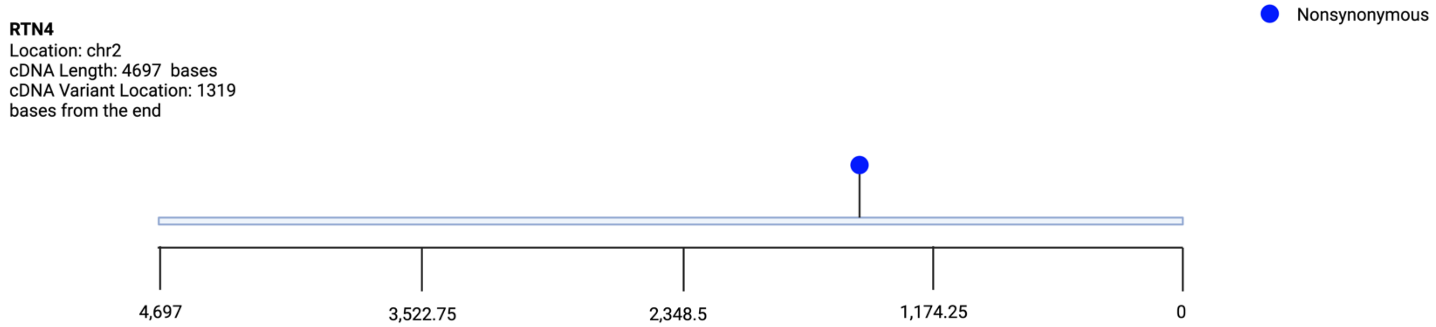

c.

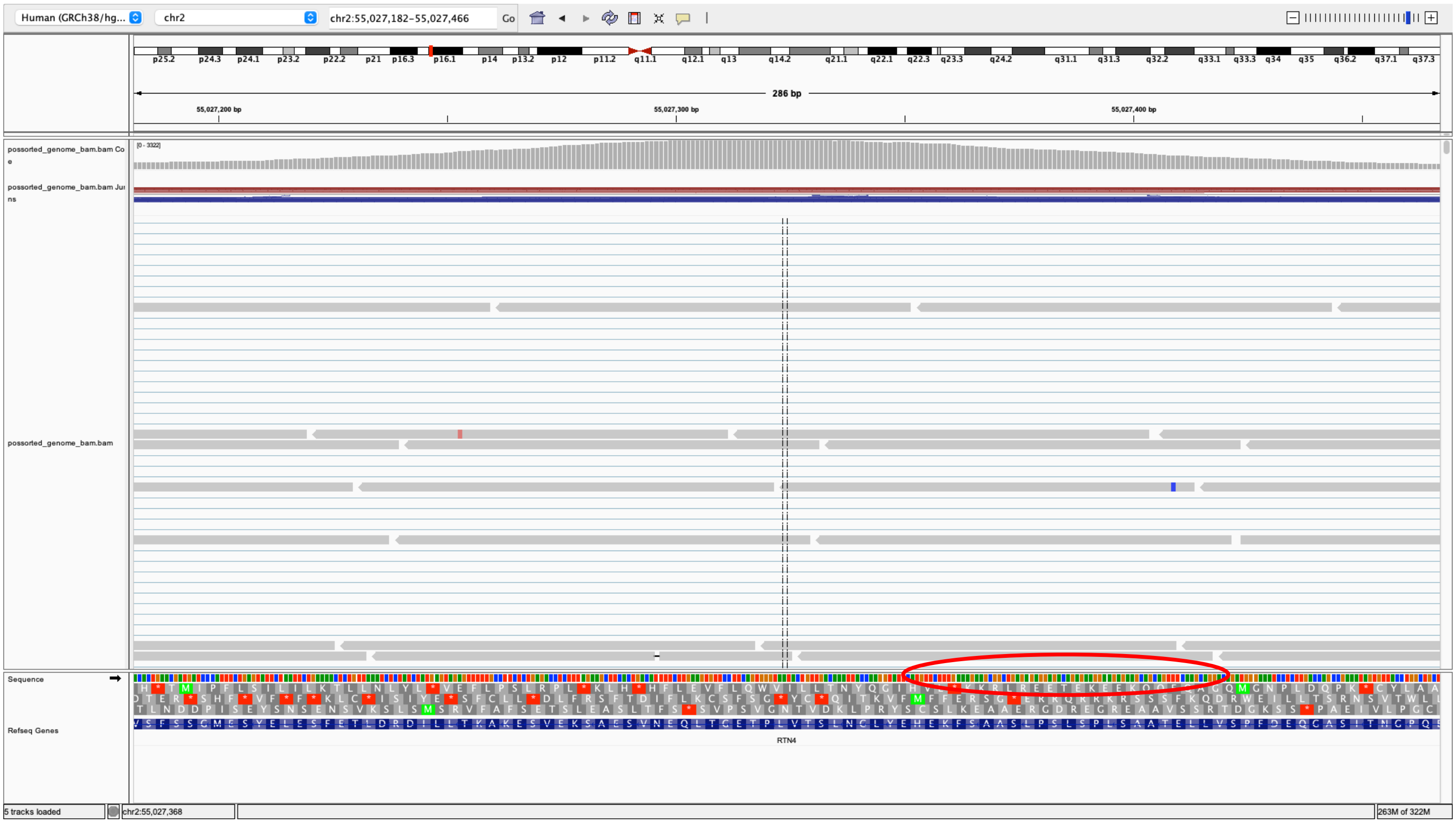

Figure S3: RTN4 variant mispriming within patient datasets. (a-b) The RTN4 variant's position in the middle of the gene, which should preclude its detection in single-nuclei 3'- or 5'-datasets. (c) Presence of A-repeat sequences might explain why this germline variant was detected in both datasets with 12-25% of cells genotyped.

Supplemental Figure 4

a.

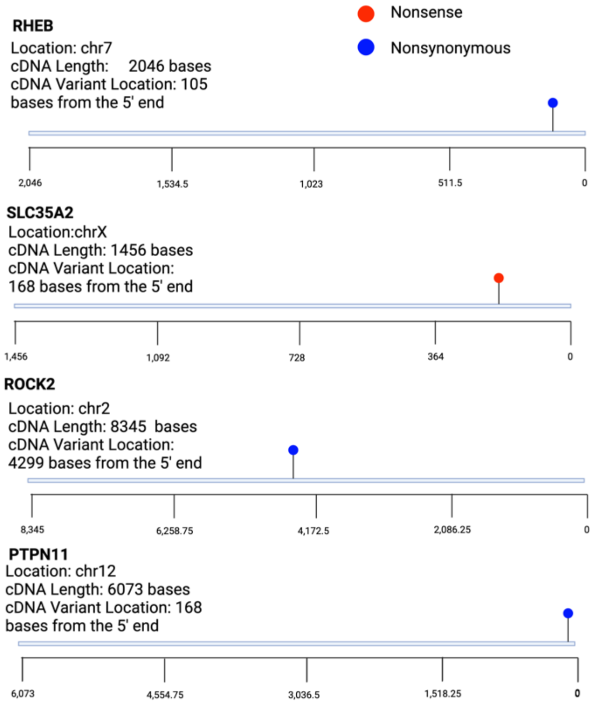

b.

		RHEB		SLC35A2		PTPN11		ROCK2	
		3'	5'	3'	5'	3'	5'	3'	5'
Number of Genotyped Calls	No Call	5984	4121	8262	7323	5455	4774	5448	5651
	Ref Call	38	1464	3	184	51	903	58	34
	Alt Call	7	87	0	5	0	8	0	0

c.

Variant	3p	5p
RHEB	7/45 = .16 = 16%	87/1551 = .05 = 5%
SLC35A2	0/3 = 0 = 0%	5/189 = .03 = 3%
PTPN11	0/51 = 0 = 0%	8/911 = .01 = 1%
ROCK2	0/58= 0 = 0%	0/34 = 0 = 0%

d.

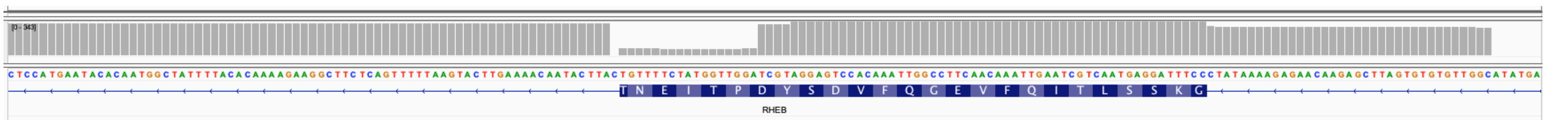

e.

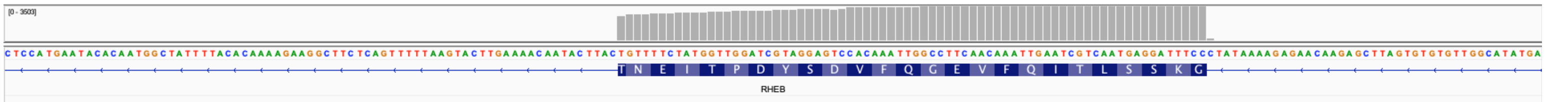

f.

		RHEB				SLC35A2				PTPN11				ROCK2			
	Cell Type	No Call	Ref	Alt	VAF	No Call	Ref	Alt	VAF	No Call	Ref	Alt	VAF	No Call	Ref	Alt	VAF
3p	Oligodendrocyte	970	5	0	0%	6655	3	0	0%	1160	6	0	0%	1164	2	0	0%
	Microglia	1621	9	0	0%	881	0	0	0%	1335	0	0	0%	1333	2	0	0%
	OPCs	1163	4	4	50%	408	0	0	0%	796	3	0	0%	797	2	0	0%
	Astrocytes	264	1	3	75%	246	0	0	0%	747	1	0	0%	746	2	0	0%
	Mitochondrial	433	2	0	0%	0	0	0	0%	99	1	0	0%	98	2	0	0%
	Excitatory Neurons	578	9	0	0%	27	0	0	0%	1009	33	0	0%	999	43	0	0%
	CGE-Derived Interneurons	320	5	0	0%	17	0	0	0%	72	2	0	0%	73	1	0	0%
	Lymphocytes	504	1	0	0%	7	0	0	0%	14	0	0	0%	14	0	0	0%
	MGE-Derived Interneurons	131	2	0	0%	21	0	0	0%	223	5	0	0%	224	4	0	0%
5p	Oligodendrocyte	20	4	3	42%	6211	175	5	3%	1089	108	4	4%	1200	1	0	0%
	Microglia	688	54	3	5%	484	0	0	0%	1379	59	0	0%	1437	1	0	0%
	OPCs	495	61	27	31%	297	4	0	0%	763	56	2	3%	820	2	0	0%
	Astrocytes	97	21	11	32%	104	1	0	0%	637	42	0	0%	678	1	0	0%
	Mitochondrial	10	0	0	0%	0	0	0	0%	293	37	1	3%	328	3	0	0%
	Excitatory Neurons	1621	894	30	3%	58	1	0	0%	396	519	1	0%	895	20	0	0%
	CGE-Derived Interneurons	573	199	5	5%	146	24	0	0%	47	24	0	0%	69	2	0	0%
	Lymphocytes	250	15	1	6%	3	0	0	0%	31	0	0	0%	31	0	0	0%
	MGE-Derived Interneurons	367	216	7	3%	20	0	0	0%	139	58	0	0%	193	4	0	0%

Figure S4: Genotyping of somatic variants. (a) Variant position relative to cDNA length for each detected somatic variant. (b) Number of genotyped cells and (c) variant allele frequency (VAF) for each somatic variant. (d-e) IGV images of snRNA-seq data from Patient 47 show the presence of repeat A sequences surrounding coverage in both the 3' (d) and 5' (e) datasets. (f) VAF frequency calculations for each somatic variant for each cell type cluster.
